# Supplementary material for: Intrinsic and extrinsic determinants of conditional localization of Mms6 to magnetosome organelles in Magnetospirillum magneticum AMB-1
Source: J Bacteriol. 2024 May 31;206(6):e00008-24. doi: 10.1128/jb.00008-24 (PMC11332177; doi:10.1128/jb.00008-24)
Supplement: Supplemental material — Supplemental results, Figures S1 to S9, and Tables S1 to S8. [file jb.00008-24-s0001.pdf]

Supplemental Materials

Intrinsic and extrinsic determinants of conditional localization of Mms6 to magnetosome organelles in *Magnetospirillum magneticum* AMB-1

Carson D. Bickley[a], Arash Komeili[a#]

[a] Department of Plant and Microbial Biology, University of California, Berkeley, California, USA

Running Title: Dynamic Mms6 localization in *M. magneticum* AMB-1

[#] Address correspondence to Arash Komeili, komeili@berkeley.edu

Supplemental Results:

Deletions causing minor effects on Mms6 localization

Other deletions that caused at most minor changes in Mms6 localization are shown in Supplementary Figure 3. Some of these small changes may be explained by biological variance and may register as significant due to increased sensitivity of chi-squared and fisher's exact tests at higher sample sizes. A deletion of AMB-1 MAI region 3, consisting of the *mamGFDC* operon and the *mms6* cluster, resulted in a small but significant increase in Mms6-Halo localized in foci under NBPC, while Mms6-Halo still localized to magnetosomes in most cells grown under BPC. In a deletion of the *mamGFDC* operon comprising *mamG, mamF*, *mamD*, and *mamC*, Mms6-Halo had a significant but slight increase in chain alignment under NBPC, while Mms6-Halo localization was unchanged in BPC. In a strain lacking *mamJ* and *limJ,* genes that regulate the function of cytoskeletal filament MamK (1,2), Mms6-Halo localized normally under NBPC and had significant but small increases in chain alignment and foci localization under BPC compared to WT cells. In a deletion of *mamK,* Mms6-Halo localized as in WT, except for a small, significant increase in foci localization in NBPC. Since MamK, MamJ, and LimJ regulate magnetosome chain organization, the changes observed may be due to variations in chain architecture. In a deletion of *mamD,* Mms6-Halo showed a small, significant increase in foci localization in NBPC and a small, significant increase in diffuse localization in BPC. Finally, in a deletion of *mamP*, Mms6-Halo showed a small, significant increase in foci localization in BPC. In addition to the ΔMAI ΔMIS mutant (Fig. 4), the localization of Mms6-GFP was examined in three other mutants deficient in magnetosome membrane formation, Δ*mamI,* Δ*mamL,* and Δ*mamN* Δ*mamO* (Supplementary Figure 4). As expected, Mms6-GFP was diffuse in these mutants regardless of biomineralization condition.

Localization determinants of MmsF

To explore the possibility that localization of the selected magnetite maturation proteins is controlled by the same factors that control Mms6 localization, the proteins were expressed in various mutant backgrounds. Whereas GFP-MmsF localized to magnetosomes regardless of biomineralization conditions in WT as previously shown, it was distributed around the inner membrane in a strain lacking the MAI and magnetosome gene islet (MIS) (Supplementary Figure 8). Similar to Mms6-Halo, GFP-MmsF fails to localize to magnetosomes in Δ*mamO* cells (Supplementary Figure 8). However, in contrast to Mms6-Halo, GFP-MmsF does not localize to magnetosomes in the absence of *mamN* (Supplementary Figure 8). These results show that while MamN inhibits Mms6 localization, it is required for MmsF localization to magnetosomes.

References

1 Scheffel, A., Gruska, M., Faivre, D., Linaroudis, A., Plitzko, J. M., & Schüler, D. 2006. An acidic protein aligns magnetosomes along a filamentous structure in magnetotactic bacteria. *Nature*, *440*(7080), 110–114. https://doi.org/10.1038/nature04382

2 Draper, O., Byrne, M. E., Li, Z., Keyhani, S., Barrozo, J. C., Jensen, G., & Komeili, A. 2011. MamK, a bacterial actin, forms dynamic filaments in vivo that are regulated by the acidic proteins MamJ and LimJ. *Molecular microbiology*, *82*(2), 342–354. https://doi.org/10.1111/j.1365-2958.2011.07815.x

Supplemental Results

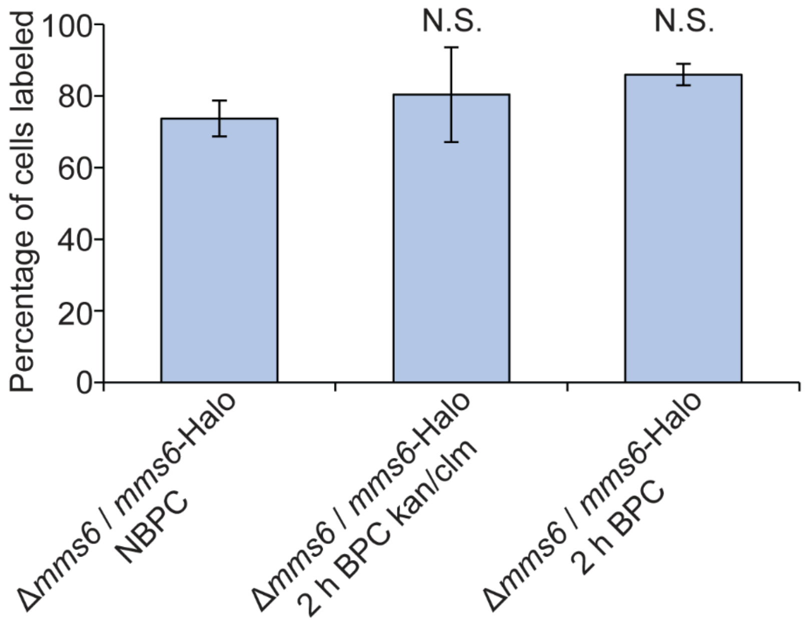

Supplementary Figure 1. Percentage of cells labeled with Mms6-Halo fluorescence before and during relocalization time course with and without antibiotics. *P* value was calculated by two tailed Student's *t* test comparing given dataset with Δ*mms6* / *mms6*-Halo NBPC (N.S. $P > 0.01$). NBPC $n = 2881$ DAPI labeled cells, 2 h BPC kan/clm $n = 1315$ DAPI labeled cells, 2 h BPC $n = 2822$ DAPI labeled cells.

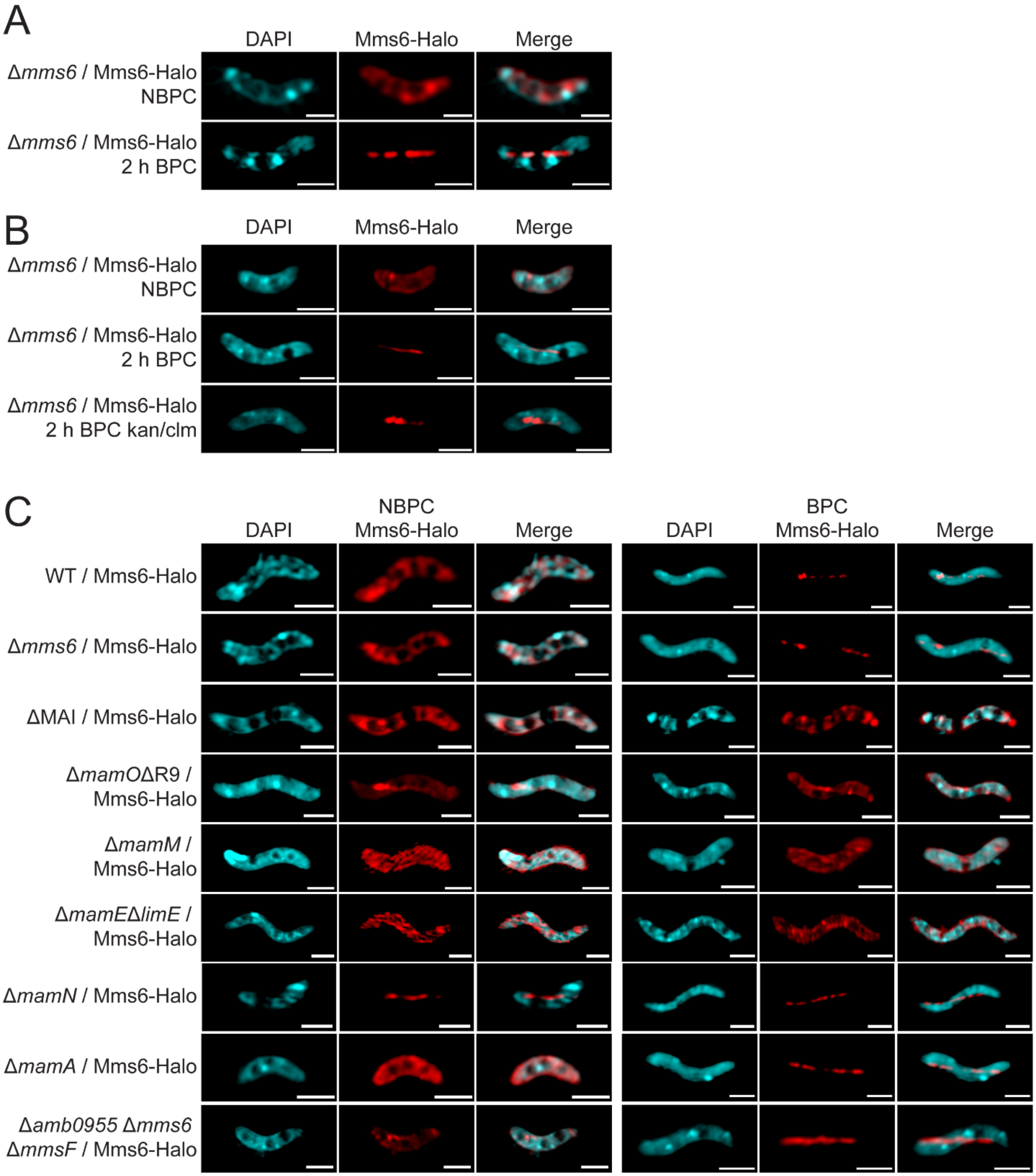

Supplementary Figure 2. Representative super resolution 3D Structured Illumination Microscopy (SIM) images of cells expressing Mms6-Halo. (A) Representative images of WT cells expressing Mms6-Halo from biomineralization time course. WT cells grown under standard growth conditions expressing Mms6-Halo shown in red and DAPI shown in blue. (B)

Representative images of WT cells expressing Mms6-Halo from biomineralization time course with and without antibiotics. WT cells grown under standard growth conditions expressing Mms6-Halo shown in red and DAPI shown in blue. Sample labeled “kan/clm” was given 700 μg/mL kanamycin and 400 μg/mL chloramphenicol to prevent the synthesis of new Mms6-Halo during and after the one-hour labeling step. Scale bars = 1 μm. (C) Representative images of cells of different mutant backgrounds expressing Mms6-Halo in red with DAPI shown in blue. Scale bars = 1 μm.

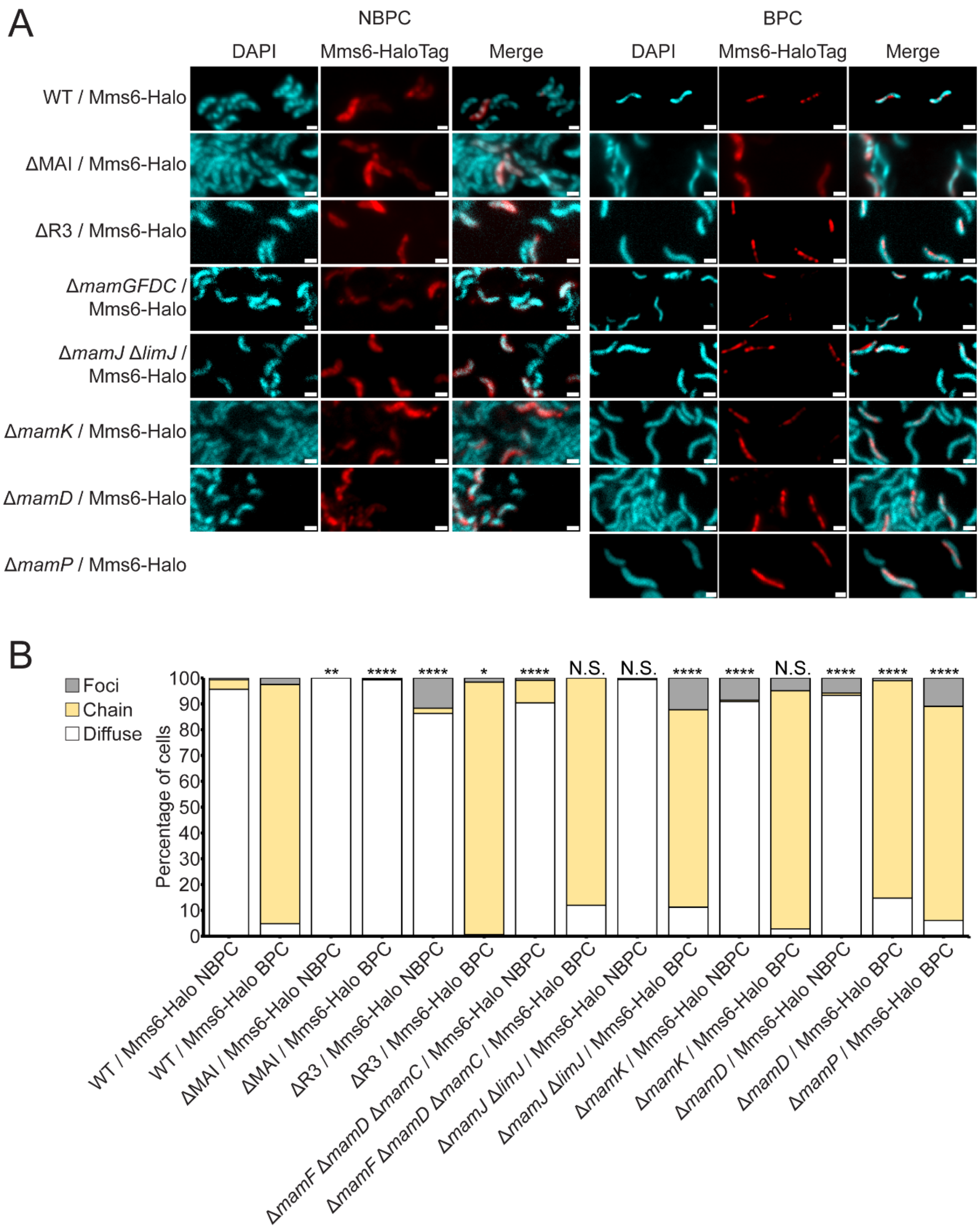

Supplementary Figure 3. Mms6-Halo localization in further MAI protein deletion backgrounds (A) Representative fluorescence microscopy images of AMB-1 with different genetic

backgrounds expressing Mms6-Halo and grown in standard conditions. JF549 HaloTag ligand fluorescence is shown in red and DAPI in blue. Scale bars = 1 μm. (B) Blind quantification of localization patterns of Mms6-Halo. *P* values were calculated by Fisher's exact test comparing given dataset to WT / *mms6*-Halo (N.S. no significant difference $P > .01$) (* = $P < .01$) (** = $P < 10^{-3}$) (**** = $P < 10^{-5}$). WT / *mms6*-Halo NBPC *n* = 1010 cells, WT / *mms6*-Halo BPC *n* = 477 cells, ΔMAI / *mms6*-Halo NBPC *n* = 262 cells, ΔMAI / *mms6*-Halo BPC *n* = 400 cells, ΔR3 / *mms6*-Halo NBPC *n* = 197 cells, ΔR3 / *mms6*-Halo BPC *n* = 316 cells, Δ*mamF* Δ*mamD* Δ*mamC* / *mms6*-Halo NBPC *n* = 996 cells, Δ*mamF* Δ*mamD* Δ*mamC* / *mms6*-Halo BPC *n* = 92 cells, Δ*mamJ* Δ*limJ* / *mms6*-Halo NBPC *n* = 160 cells, Δ*mamJ* Δ*limJ* / *mms6*-Halo BPC *n* = 277 cells, Δ*mamK* / *mms6*-Halo NBPC *n* = 175 cells, Δ*mamK* / *mms6*-Halo BPC *n* = 142 cells, Δ*mamD* / *mms6*-Halo NBPC *n* = 223 cells, Δ*mamD* / *mms6*-Halo BPC *n* = 197 cells, Δ*mamP* / *mms6*-Halo BPC *n* = 483 cells.

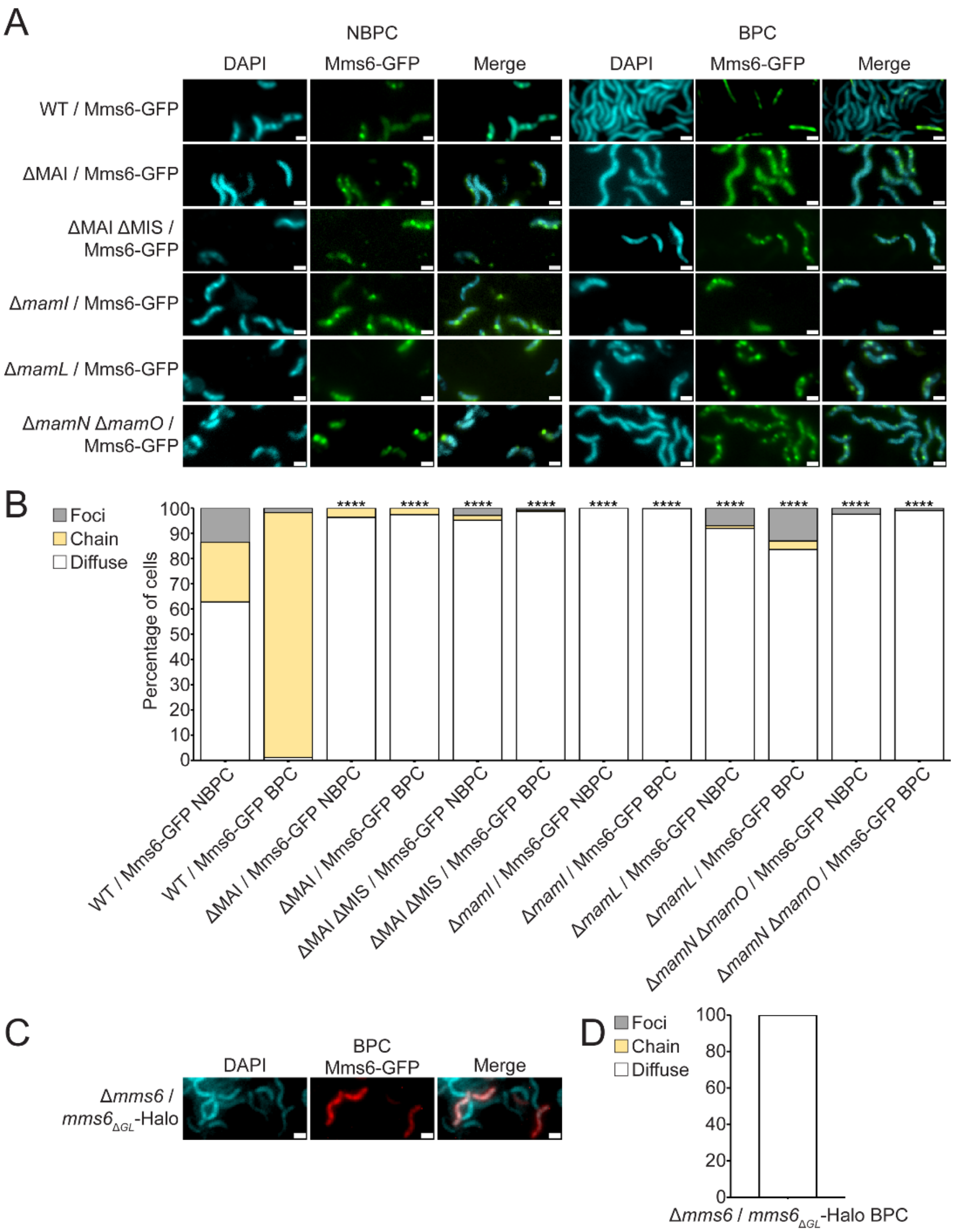

Supplementary Figure 4. Mms6-GFP localization in further MAI protein deletion backgrounds (A) Representative fluorescence microscopy images of AMB-1 with different genetic backgrounds expressing Mms6-GFP and grown in standard conditions. GFP is shown in green and DAPI in blue. Scale bars = 1 µm. (B) Blind quantification of localization patterns of Mms6-GFP. *P* values were calculated by Fisher's exact test comparing given dataset to WT / *mms6*-GFP (**** = $P < 10^{-5}$). WT / *mms6*-GFP NBPC n = 199 cells, WT / *mms6*-GFP BPC n = 282 cells, ΔMAI / *mms6*-GFP NBPC n = 356 cells, ΔMAI / *mms6*-GFP BPC n = 1070 cells, ΔMAI ΔMIS / *mms6*-GFP NBPC n = 104 cells, ΔMAI ΔMIS / *mms6*-GFP BPC n = 540 cells, Δ*mamI* / *mms6*-GFP NBPC n = 72 cells, Δ*mamI* / *mms6*-GFP BPC n = 77 cells, Δ*mamL* / *mms6*-GFP NBPC n = 487 cells, Δ*mamL* / *mms6*-GFP BPC n = 207 cells, Δ*mamN* Δ*mamO* / *mms6*-GFP NBPC n = 82 cells, Δ*mamN* Δ*mamO* / *mms6*-GFP BPC n = 715 cells. (C) Representative fluorescence

microscopy images of AMB-1 strain expressing Δ*mms6* / $mms6_{\Delta GL}$-Halo BPC and grown in standard conditions. JF549 HaloTag ligand fluorescence is shown in red and DAPI in blue. Scale bars = 1 µm. (D) Quantification of localization patterns of Mms6-Halo. Δ*mms6* / $mms6_{\Delta GL}$-Halo BPC *n* = 3541 cells.

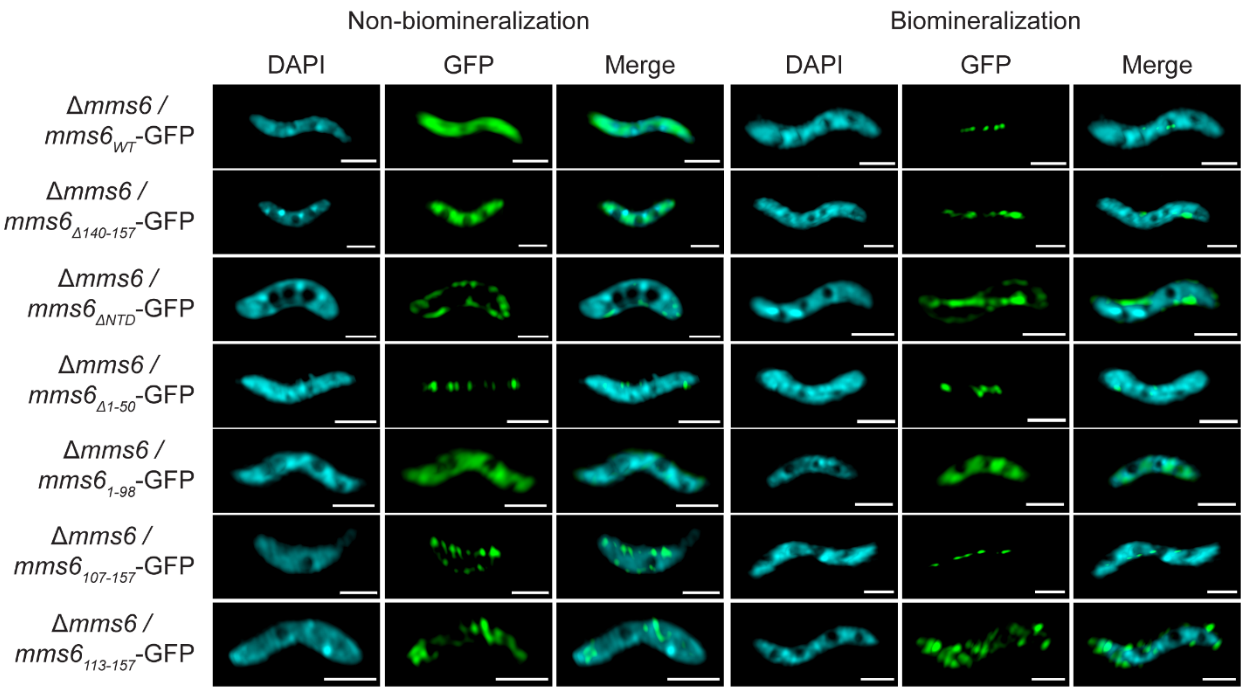

Supplementary Figure 5. Representative super resolution 3D Structured Illumination Microscopy (SIM) images of cells expressing Mms6-GFP. Representative images of Δ*mms6* mutant cells expressing Mms6-GFP or a mutant Mms6 protein tagged C-terminally with GFP. GFP is shown in green and DAPI in blue. Scale bars = 1 μm.

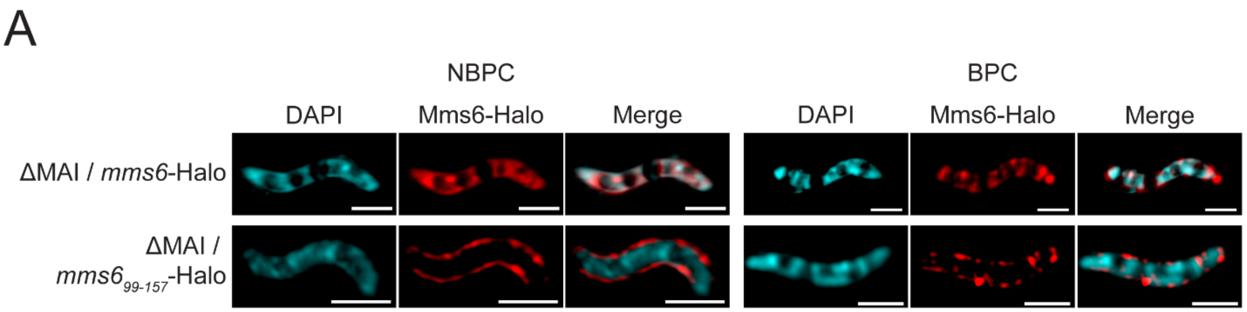

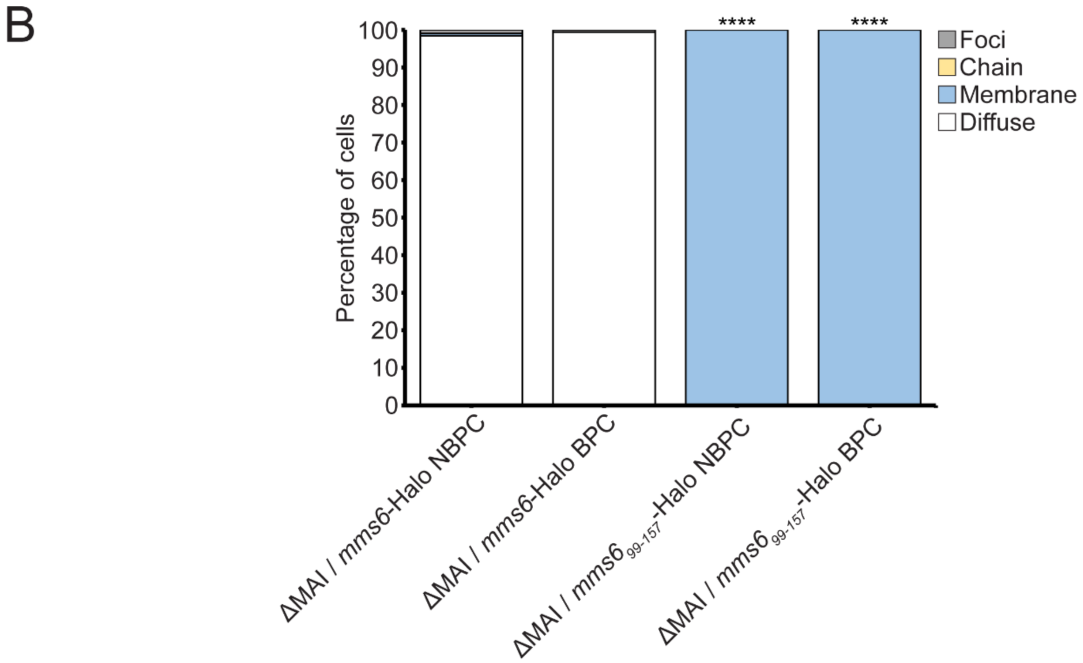

Supplementary Figure 6. Mms6$_{99\text{-}157}$-Halo does not require other magnetosome proteins to translocate into membranes. (A) Representative images of ΔMAI mutant cells expressing Mms6-Halo or Mms6$_{99\text{-}157}$-Halo shown in red. DAPI counterstain is shown in blue. Scale bars = 1 µm. (B) Blind quantification of localization patterns of given protein. *P* values were calculated by Fisher's exact test comparing given dataset to ΔMAI / *mms6*-Halo grown in matching biomineralization condition (**** = $P < 10^{-5}$). ΔMAI / *mms6*-Halo NBPC n = 318 cells, ΔMAI / *mms6*-Halo BPC n = 174 cells, ΔMAI / *mms6*$_{99\text{-}157}$-Halo NBPC n = 82 cells, ΔMAI / *mms6*$_{99\text{-}157}$-Halo BPC n = 23 cells.

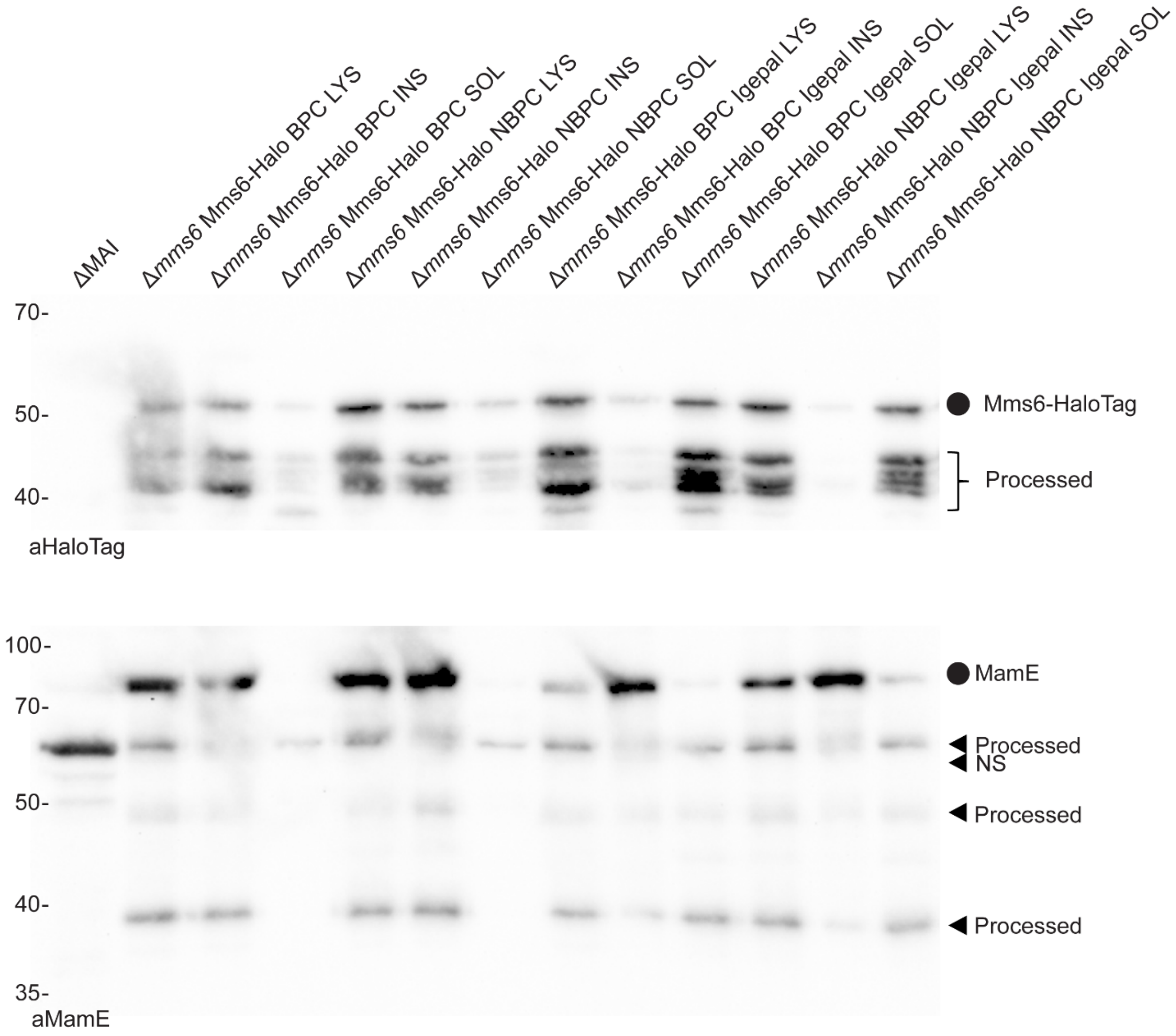

Supplementary Figure 7. Mms6 is weakly associated with insoluble cell contents. Immunoblotting analysis of cell fractions after fractionation of cells expressing Mms6-Halo. HaloTag and MamE were probed for in the whole-cell lysate (LYS) before centrifugation, as well as in insoluble (INS) and soluble (SOL) fractions afterwards. The cell fractionation was performed with and without 0.4% Igepal. Full length protein bands are marked with circles, and both processed fragments and non-specific bands (NS) are marked with arrows.

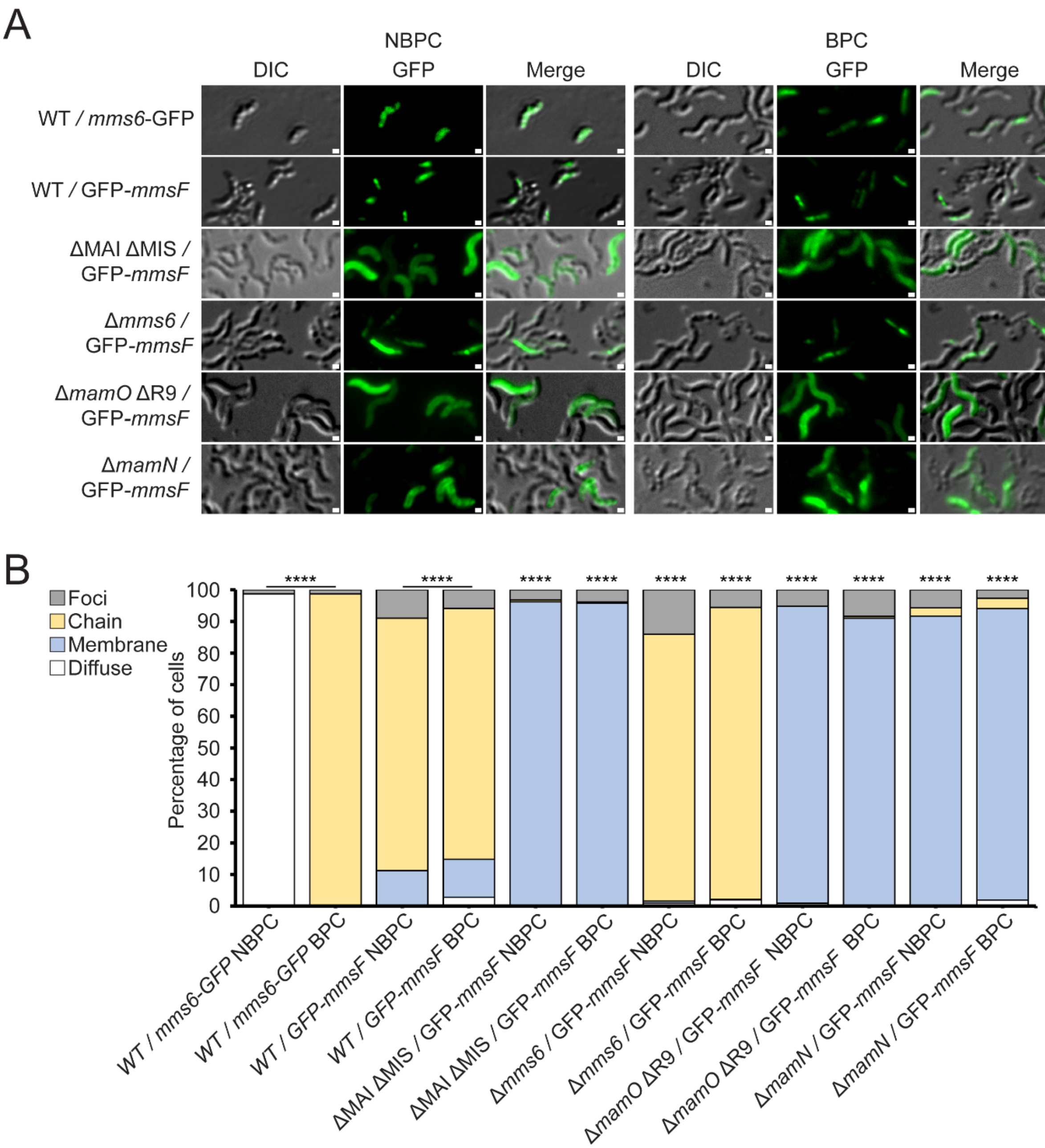

Supplementary Figure 8. MmsF requires *mamN* and *mamO* for magnetosome localization. (A) Representative fluorescence microscopy images of WT or mutant AMB-1 cells grown under standard growth conditions expressing Mms6 or MmsF GFP fusions. GFP is shown in green and transmitted light (TL) is displayed to show outlines of AMB-1 cells. Scale bars = 1 µm. (B) Blind quantification of localization patterns of GFP tagged magnetosome proteins *in vivo* expressed in either WT or *Δmms6* cells. The Y-axis represents percentage of total cell count with indicated protein fluorescence pattern. *P* values were calculated by Fisher's exact test comparing indicated datasets (**** $P < 10^{-5}$). WT / *mms6*-GFP NBPC *n* = 1074 cells, WT / *mms6*-GFP BPC *n* = 1317 cells, WT / GFP-*mmsF* NBPC *n* = 2412 cells, WT / GFP-*mmsF* BPC *n* = 1295 cells, ΔMAI ΔMIS / GFP-*mmsF* NBPC *n* = 1034 cells, ΔMAI ΔMIS / GFP-*mmsF* BPC *n* = 519 cells,

Δmms6 / GFP-*mmsF* NBPC *n* = 497 cells, *Δmms6* / GFP-*mmsF* BPC *n* = 1062 cells, Δ*mamO* ΔR9 / GFP-*mmsF* NBPC *n* = 478 cells, Δ*mamO* ΔR9 / GFP-*mmsF* BPC *n* = 91 cells, Δ*mamN* / GFP-*mmsF* NBPC *n* = 421 cells, Δ*mamN* / GFP-*mmsF* BPC *n* = 524 cells.

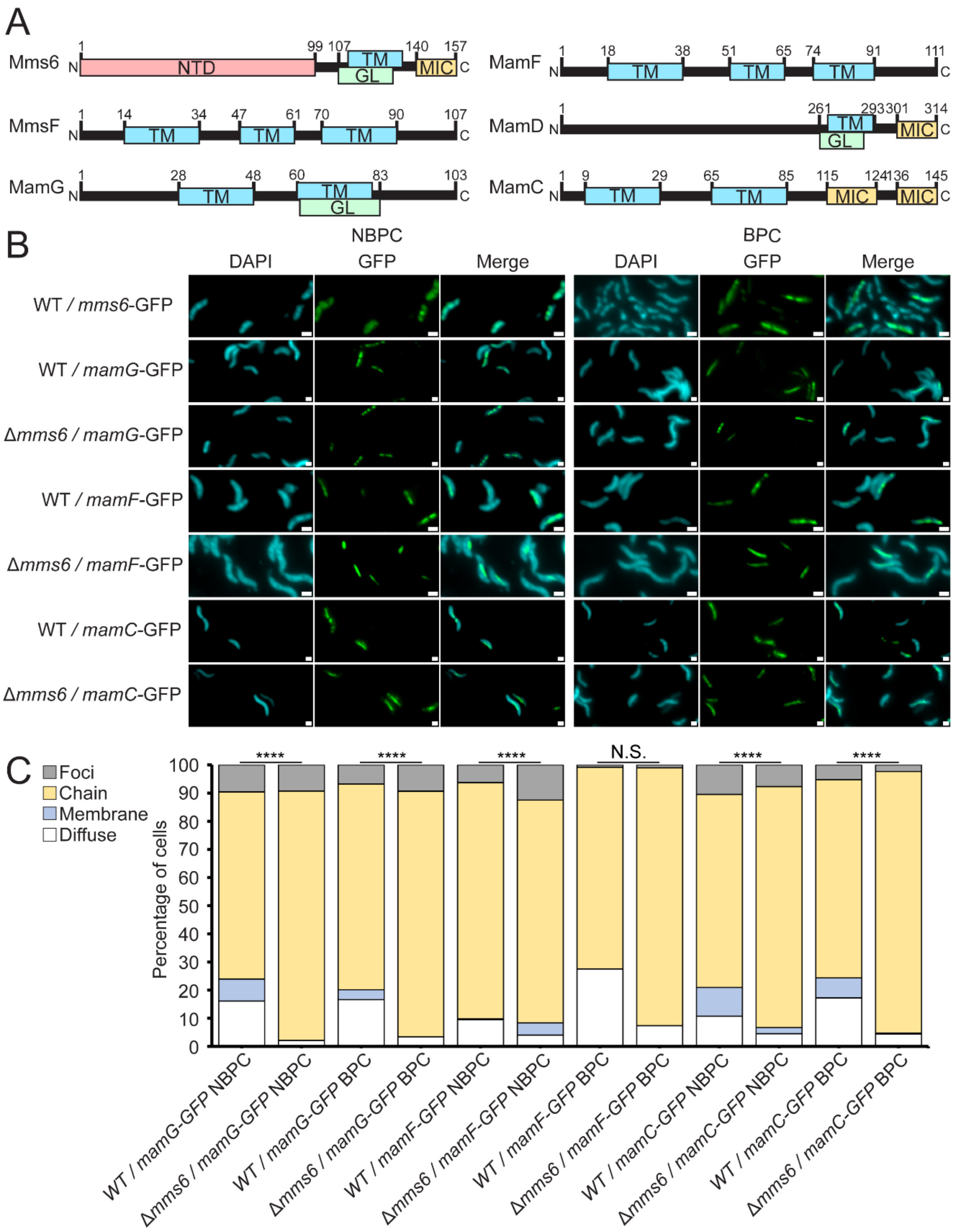

Supplementary Figure 9. Magnetite shaping proteins do not require *mms6* for magnetosome localization. (A) Structural features of several magnetosome proteins implicated in crystal maturation. N-terminal domains (NTD), glycine leucine repeat domains (GL), transmembrane domains (TM), magnetite-interacting component (MIC). (B) Representative fluorescence microscopy images of WT or Δ*mms6* AMB-1 cells grown under standard growth conditions

expressing magnetosome protein GFP fusions. GFP is shown in green, and DAPI in blue. Scale bars = 1 µm. (C) Blind quantification of localization patterns of GFP tagged magnetosome proteins *in vivo* expressed in WT or Δ*mms6* cells. The Y-axis represents percentage of total cell count with indicated protein fluorescence pattern. *P* values were calculated by Fisher's exact test comparing indicated datasets (N.S. no significant difference $P > .01$) (**** $P < 10^{-5}$). WT / *mamG*-GFP NBPC *n* = 1116 cells, *Δmms6* / *mamG*-GFP NBPC *n* = 1440 cells, WT / *mamG*-GFP BPC *n* = 367 cells, *Δmms6* / *mamG*-GFP BPC *n* = 1560 cells, WT / *mamF*-GFP NBPC *n* = 1238 cells, *Δmms6* / *mamF*-GFP NBPC *n* = 1583 cells, WT / *mamF*-GFP BPC *n* = 1784 cells, *Δmms6* / *mamF*-GFP BPC *n* = 2381 cells, WT / *mamC*-GFP NBPC *n* = 372 cells, *Δmms6* / *mamC*-GFP NBPC *n* = 793 cells, WT / *mamC*-GFP BPC *n* = 1253 cells, *Δmms6* / *mamC*-GFP BPC *n* = 1881 cells.

Supplemental Tables:

Strain	Description	Reference
AK30	AMB-1 Wildtype	12
AK31	ΔMAI	37
AK33	Δ*mamL*	37
AK36	ΔR3	37
AK44	Δ*mamM*	37
AK55	Δ*mamN*	37
AK64	Δ*mamI*	37
AK79	Δ*mamA*	19
AK82	Δ*mamK*	46
AK94	Δ*mamO* and deletion of MAI region 9 (R9)	88
AK96	Δ*mamE* Δ*limE*	88
AK103	Δ*mms6*	64
AK104	Δ*mmsF*	64
AK108	Δ*mamJ* Δ*limJ*	47
AK109	Δ*mamF* Δ*mamD* Δ*mamC*	37
AK124	Δ*amb0955* Δ*mms6* Δ*mmsF*	37
AK272	ΔMAI and deletion of magnetosome islet in AMB-1 WT	50
AK329	Δ*mamN* Δ*mamO*	This publication

Supplementary Table S1. *Magnetospirillum* strains used in this study.

Plasmid	Description	Plasmid backbone	Reference
pAK272	Ptac-*mamI* -GFP	pAK22	50
pAK452	Ptac-*mamC* -GFP	pAK22	88
pAK454	Ptac-*mamF* -GFP	pAK22	88
pAK532	Ptac-GFP-*mmsF*	pAK22	64
pAK976	Ptac-mHaloTag	pAK22	84
pAK1101	Ptac-*mms6* -HaloTag	pAK22	50
pAK1102	Ptac-*mms6* -GFP	pAK22	50
pAK1204	Ptac-*mamD* -GFP	pAK22	This publication
pAK1440	Ptac-*mamG* -GFP	pAK22	This publication
pAK1441	Ptac-*mms6* $_{51\text{-}157}$ -GFP	pAK1102	This publication
pAK1443	Ptac-*mms6* $_{1\text{-}139}$ -GFP	pAK1102	This publication
pAK1444	Ptac-*mms6* $_{1\text{-}98}$ -GFP	pAK1102	This publication
pAK1445	Ptac-*mms6* $_{107\text{-}157}$ -GFP	pAK1102	This publication
pAK1446	Ptac-*mms6* $_{113\text{-}157}$ -GFP	pAK1102	This publication
pAK1447	Ptac-GFP-*mms6* $_{\mathrm{NTD}}$*mmsF*	pAK532	This publication
pAK1456	Ptac-*mms6* $_{99\text{-}157}$-GFP	pAK1102	This publication

Supplementary Table S2. Plasmids used in this study.

Primer	Target	Sequence
CB01	pAK-*mms6* $_{1\text{-}98}$-GFP	GATAACAATTTCACACAGGAAACAGAATTCATGCCAGCTCAGATCGCCAACGGAGTTATT
CB02	pAK-*mms6* $_{1\text{-}98}$-GFP	TTCTTCTCCTTTACTCATGGATCCGACGACCTTGGCACCGGCGGC
CB03	pAK-*mms6* $_{99\text{-}157}$-GFP	CAATTTCACACAGGAAACAGAATTCATGGGAACCATCTGGACCGGTAAGGGGCTG
CB04	pAK-*mms6* $_{99\text{-}157}$-GFP	GAAAAGTTCTTCTCCTTTACTCATGGATCCGGCCAGCGCGTCGCGCAGTTCGACTTC
CB05	pAK-*mms6* $_{51\text{-}157}$-GFP	GATAACAATTTCACACAGGAAACAGAATTCATGGTCGCCAAGACCGGCATCGCCGCCAAG
CB06	pAK-*mms6* $_{51\text{-}157}$-GFP	GAAAAGTTCTTCTCCTTTACTCATGGATCCGGCCAGCGCGTCGCGCAGTTCGACTTCCTC
CB07	pAK-*mms6* $_{1\text{-}139}$-GFP	GATAACAATTTCACACAGGAAACAGAATTCATGCCAGCTCAGATCGCCAACGGAGTTATT
CB08	pAK-*mms6* $_{1\text{-}139}$-GFP	GAAAAGTTCTTCTCCTTTACTCATGGATCCACGGCTCTTCATATACGCGTAAACCGCCCC
CB09	pAK-*mms6* $_{107\text{-}157}$-gfp	GATAACAATTTCACACAGGAAACAGAATTCATGGGGCTGGGCCTCGGTCTGGGTCTCGGT
CB10	pAK-*mms6* $_{107\text{-}157}$-gfp	GAAAAGTTCTTCTCCTTTACTCATGGATCCGGCCAGCGCGTCGCGCAGTTCGACTTCCTC
CB11	pAK-*mms6* $_{113\text{-}157}$-gfp	GATAACAATTTCACACAGGAAACAGAATTCATGGGTCTCGGTCTGGGCGCGTGGGGGCCG
CB12	pAK-*mms6* $_{113\text{-}157}$-gfp	GAAAAGTTCTTCTCCTTTACTCATGGATCCATACGCGTAAACCGCCCCGGCGCCAACAAC
CB13	pAK-GFP-*mms6* $_{NTD}$*mmsF*	GAAGCGGCGGCCAAAGCAGCAGCGGGATCCCCAGCTCAGATCGCCAACGGAGTTATTTGC
CB14	pAK-GFP-*mms6* $_{NTD}$*mmsF*	GCTGCGAAGGATAGCTTCAGTGGATCCGACGACCTTGGCACCGGCGGCGGCCTTG
CB15	Ptac-*mamG*-GFP	GATAACAATTTCACACAGGAAACAGAATTCATGGCCGCTCAGGTTGGAGGGCAGATTTTG
CB16	Ptac-*mamG*-GFP	GAAAAGTTCTTCTCCTTTACTCATGGATCCAGTCGCATCGGCGGCGGCATCAACCTC

Supplementary Table S3. Primers used in this study.

Figure	Dataset/strain	Compared datasets/strains	Chi-square test of independence		Cramer's V effect size		
			P-value	Significance	V	df	Effect Size
Figure 1B	NBPC	30 min BPC	0.3517	NS	0.0422	3	Negligible
	NBPC	1 h BPC	$< 10^{-5}$	****	0.3162	3	Large
	NBPC	1.5 h BPC	$< 10^{-5}$	****	0.3496	3	Large
	NBPC	2 h BPC	$< 10^{-5}$	****	0.4602	3	Large
Figure 2F	NBPC	2 h BPC	$< 10^{-5}$	****	0.5654	2	Large

Supplementary Table S4. Statistical tests using the Chi-square test of independence. The Chi-square test of independence tests the hypothesis that two variables are unrelated. Cramer's *V* is an effect size measurement that measures how strongly two categorical fields are associated.

Figure	Dataset/strain	Compared datasets/strains	Mann-Whitney U Test	
			p-value	Significance
Figure 1C	Δ*mms6* / empty vector	Δ*mms6* / *mms6*-GFP	0.000605172	**
	Δ*mms6* / empty vector	Δ*mms6* / *mms6*-Halo	6.92669E-05	***
Figure 6B	Δ*mms6* / empty vector	Δ*mms6* / *mms6*-GFP	0.000605172	**
	Δ*mms6* / empty vector	Δ*mms6* / *mms6*-Halo	6.92669E-05	***
	Δ*mms6* / empty vector	Δ*mms6* / $mms6_{1\text{-}139}$-GFP	0.000129814	**
	Δ*mms6* / empty vector	Δ*mms6* / $mms6_{99\text{-}157}$-GFP	0.00125393	*
	Δ*mms6* / empty vector	Δ*mms6* / $mms6_{51\text{-}157}$-GFP	0.000743289	**
	Δ*mms6* / empty vector	Δ*mms6* / $mms6_{1\text{-}98}$-GFP	0.518827467	NS
	Δ*mms6* / empty vector	Δ*mms6* / $mms6_{113\text{-}157}$-GFP	0.00133003	*
	Δ*mms6* / empty vector	Δ*mms6* / $mms6_{107\text{-}157}$-GFP	0.030961701	NS

Supplementary Table S5. Statistical tests using the Mann Whitney *U* test. The Mann Whitney *U* test is a non-parametric test for the null hypothesis that the means of two populations are equal.

Figure	Dataset/strain	Compared datasets/strains	Fisher's Exact Test		Cramer's V effect size		
			P-value	Significance	V	df	Effect Size
Figure 3D	NBPC	Δ*mms6* / *mms6*-Halo 2 h BPC kan/clm	$< 10^{-5}$	****	0.708	2	Large
	NBPC	Δ*mms6* / *mms6*-Halo 2 h BPC	$< 10^{-5}$	****	0.8382	2	Large
Figure 4B	ΔMAI ΔMIS / *mms6*-GFP NBPC	ΔMAI ΔMIS / GFP-*mmsF* NBPC	$< 10^{-5}$	****	0.9646	3	Large
	ΔMAI ΔMIS / *mms6*-GFP BPC	ΔMAI ΔMIS / GFP-*mmsF* BPC	$< 10^{-5}$	****	0.8411	3	Large
Figure 5B	WT / *mms6*-Halo NBPC	Δ*mms6* / *mms6*-Halo NBPC	0.1629	NS	0.05754	2	Negligible
	WT / *mms6*-Halo BPC	Δ*mms6* / *mms6*-Halo BPC	0.1828	NS	0.03707	2	Negligible
	WT / *mms6*-Halo NBPC	ΔMAI / *mms6*-Halo NBPC	$< 10^{-5}$	****	0.2387	2	Medium
	WT / *mms6*-Halo BPC	ΔMAI / *mms6*-Halo BPC	$< 10^{-5}$	****	0.9243	3	Large
	WT / *mms6*-Halo NBPC	Δ*mamN* / *mms6*-Halo NBPC	$< 10^{-5}$	****	0.5439	3	Large
	WT / *mms6*-Halo BPC	Δ*mamN* / *mms6*-Halo BPC	$< 10^{-5}$	****	0.1856	3	Medium
	WT / *mms6*-Halo NBPC	Δ*mamO* ΔR9 / *mms6*-Halo NBPC	$< 10^{-5}$	****	0.3125	2	Medium
	WT / *mms6*-Halo BPC	Δ*mamO* ΔR9 / *mms6*-Halo BPC	$< 10^{-5}$	****	0.9166	3	Large
	WT / *mms6*-Halo NBPC	Δ*mamM* / *mms6*-Halo NBPC	$< 10^{-5}$	****	0.3163	2	Medium
	WT / *mms6*-Halo BPC	Δ*mamM* / *mms6*-Halo BPC	$< 10^{-5}$	****	0.7984	2	Large
	WT / *mms6*-Halo NBPC	Δ*mamE* Δ*limE* / *mms6*-Halo NBPC	$< 10^{-5}$	****	0.2779	2	Medium
	WT / *mms6*-Halo BPC	Δ*mamE* Δ*limE* / *mms6*-Halo BPC	$< 10^{-5}$	****	0.9043	2	Large
	WT / *mms6*-Halo NBPC	Δ*mamA* / *mms6*-Halo NBPC	0.1676	NS	0.1606	3	Small
	WT / *mms6*-Halo BPC	Δ*mamA* / *mms6*-Halo BPC	0.0007271	**	0.2545	2	Medium
	WT / *mms6*-Halo NBPC	Δ*amb0955* Δ*mms6* Δ*mmsF* / *mms6*-Halo NBPC	$< 10^{-5}$	****	0.4878	3	Large
	WT / *mms6*-Halo BPC	Δ*amb0955* Δ*mms6* Δ*mmsF* / *mms6*-Halo BPC	0.8531	NS	0.05436	2	Negligible
Figure 6D	Δ*mms6* / *mms6*-GFP NBPC	Δ*mms6* / *mms6*$_{1-139}$-GFP NBPC	0.0208	NS	0.2026	3	Medium
	Δ*mms6* / *mms6*-GFP BPC	Δ*mms6* / *mms6*$_{1-139}$-GFP BPC	0.0010	*	0.2223	2	Medium
	Δ*mms6* / *mms6*-GFP NBPC	Δ*mms6* / *mms6*$_{99-157}$-GFP NBPC	$< 10^{-5}$	****	0.9492	3	Large
	Δ*mms6* / *mms6*-GFP BPC	Δ*mms6* / *mms6*$_{99-157}$-GFP BPC	$< 10^{-5}$	****	0.452	3	Large
	Δ*mms6* / *mms6*-GFP NBPC	Δ*mms6* / *mms6*$_{51-157}$-GFP NBPC	$< 10^{-5}$	****	0.8187	3	Large
	Δ*mms6* / *mms6*-GFP BPC	Δ*mms6* / *mms6*$_{51-157}$-GFP BPC	0.3692	NS	0.0525	2	Negligible
	Δ*mms6* / *mms6*-GFP NBPC	*Δmms6 / mms6*$_{1-98}$*-GFP NBPC*	$< 10^{-5}$	****	0.4793	3	Large
	Δ*mms6* / *mms6*-GFP BPC	*Δmms6 / mms6*$_{1-98}$*-GFP BPC*	$< 10^{-5}$	****	0.9894	2	Large
	Δ*mms6* / *mms6*-GFP NBPC	*Δmms6 / mms6*$_{113-157}$*-GFP NBPC*	$< 10^{-5}$	****	0.4689	3	Large
	Δ*mms6* / *mms6*-GFP BPC	*Δmms6 / mms6*$_{113-157}$*-GFP BPC*	$< 10^{-5}$	****	0.9894	2	Large
	Δ*mms6* / *mms6*-GFP NBPC	*Δmms6 / mms6*$_{107-157}$*-GFP NBPC*	$< 10^{-5}$	****	0.9983	3	Large
	Δ*mms6* / *mms6*-GFP BPC	*Δmms6 / mms6*$_{107-157}$*-GFP BPC*	0.001171	*	0.2525	3	Medium
Figure 7C	WT / GFP-*mmsF* NBPC	WT / GFP-*mms6*$_{NTD}$*mmsF* NBPC	$< 10^{-5}$	****	0.7313	3	Large
	WT / GFP-*mmsF* BPC	WT / GFP-*mms6*$_{NTD}$*mmsF* BPC	0.4295	NS	0.1129	3	Small
Figure 8B	WT / *mms6*-GFP NBPC	WT / *mms6*-GFP BPC	$< 10^{-5}$	****	0.6677	2	Large
	WT / *mamD*-GFP NBPC	WT / *mamD*-GFP BPC	$< 10^{-5}$	****	0.7168	3	Large
	WT / *mamD*-GFP NBPC	ΔMAI ΔMIS / *mamD*-GFP NBPC	$< 10^{-5}$	****	0.1213	3	Small
	WT / *mamD*-GFP BPC	ΔMAI ΔMIS / *mamD*-GFP BPC	$< 10^{-5}$	****	0.8479	3	Large
	WT / *mamD*-GFP NBPC	Δ*mms6* / *mamD*-GFP NBPC	$< 10^{-5}$	****	0.1562	3	Small
	WT / *mamC*-GFP BPC	Δ*mms6* / *mamD*-GFP BPC	0.0047	*	0.06711	3	Small
	WT / *mamD*-GFP NBPC	Δ*mamO* ΔR9 / *mamD*-GFP NBPC	$< 10^{-5}$	****	0.2007	3	Medium
	WT / *mamD*-GFP BPC	Δ*mamO* ΔR9 / *mamD*-GFP BPC	$< 10^{-5}$	****	0.8989	3	Large
	WT / *mamD*-GFP NBPC	Δ*mamN* / *mamD*-GFP NBPC	$< 10^{-5}$	****	0.4502	3	Large
	WT / *mamD*-GFP BPC	Δ*mamN* / *mamD*-GFP BPC	$< 10^{-5}$	****	0.1291	3	Small

Supplementary Table S6. Statistical tests using the Fisher's exact test. Fisher's exact test tests the same hypothesis as the chi-squared test of independence but is more accurate for sample sizes under 500 and in cases where at least one sample has a value of zero. Cramer's V is an effect size measurement that measures how strongly two categorical fields are associated.

Figure	Dataset/strain	Compared datasets/strains	Jarque-Bera test for normality		Student's T test	
			p-value	Normality	*p*-value	Significance
Figure 2A	*Δmms6* / *mms6*-Halo NBPC		0.528558395	Normal		
	Δmms6 / *mms6*-Halo NBPC	*Δmms6* / *mms6*-Halo 2h BPC	0.934841967	Normal	0.28150704	N.S.
Supplementary Figure S1	*Δmms6* / *mms6*-Halo NBPC		0.453259764	Normal		
	Δmms6 / *mms6*-Halo NBPC	*Δmms6* / *mms6*-Halo 2h BPC kan/clm	0.746400935	Normal	0.48342911	N.S.
	Δmms6 / *mms6*-Halo NBPC	*Δmms6* / *mms6*-Halo 2h BPC	0.258554063	Normal	0.01430203	N.S.

Supplementary Table S7. Statistical tests using two-tailed Student's *t* test. The Student's *t* test is a parametric test that tests the difference between the mean of two samples

Figure	Dataset/strain	Compared datasets/strains	Fisher's Exact Test		Cramer's V effect size		
			P-value	Significance	V	df	Effect Size
Supplementary Figure S3B	WT / *mms6*-Halo NBPC	ΔMAI / *mms6*-Halo NBPC	0.0006087	**	0.09641	3	Small
	WT / *mms6*-Halo BPC	ΔMAI / *mms6*-Halo BPC	$< 10^{-5}$	****	0.9416	2	Large
	WT / *mms6*-Halo NBPC	ΔR3 / *mms6*-Halo NBPC	$< 10^{-5}$	****	0.2689	3	Medium
	WT / *mms6*-Halo BPC	ΔR3 / *mms6*-Halo BPC	0.001183	*	0.1224	2	Small
	WT / *mms6*-Halo NBPC	Δ*mamF* Δ*mamD* Δ*mamC* / *mms6*-Halo NBPC	$< 10^{-5}$	****	0.1094	3	Small
	WT / *mms6*-Halo BPC	Δ*mamF* Δ*mamD* Δ*mamC* / *mms6*-Halo BPC	0.0159	NS	0.1262	2	Small
	WT / *mms6*-Halo NBPC	Δ*mamJ* Δ*limJ* / *mms6*-Halo NBPC	0.03176	NS	0.07292	3	Small
	WT / *mms6*-Halo BPC	Δ*mamJ* Δ*limJ* / *mms6*-Halo BPC	$< 10^{-5}$	****	0.2384	3	Medium
	WT / *mms6*-Halo NBPC	Δ*mamK* / *mms6*-Halo NBPC	$< 10^{-5}$	****	0.2223	3	Medium
	WT / *mms6*-Halo BPC	Δ*mamK* / *mms6*-Halo BPC	0.2331	NS	0.07063	2	Small
	WT / *mms6*-Halo NBPC	Δ*mamD* / *mms6*-Halo NBPC	$< 10^{-5}$	****	0.1738	3	Medium
	WT / *mms6*-Halo BPC	Δ*mamD* / *mms6*-Halo BPC	$< 10^{-5}$	****	0.1736	2	Small
	WT / *mms6*-Halo BPC	Δ*mamP* / *mms6*-Halo BPC	$< 10^{-5}$	****	0.1752	2	Small
Supplementary Figure S4B	WT / *mms6*-GFP NBPC	ΔMAI / *mms6*-GFP NBPC	$< 10^{-5}$	****	0.5246	2	Large
	WT / *mms6*-GFP BPC	ΔMAI / *mms6*-GFP BPC	$< 10^{-5}$	****	0.9888	2	Large
	WT / *mms6*-GFP NBPC	ΔMAI ΔMIS / *mms6*-GFP NBPC	$< 10^{-5}$	****	0.3515	2	Large
	WT / *mms6*-GFP BPC	ΔMAI ΔMIS / *mms6*-GFP BPC	$< 10^{-5}$	****	0.9777	2	Large
	WT / *mms6*-GFP NBPC	Δ*mamI* / *mms6*-GFP NBPC	$< 10^{-5}$	****	0.2313	2	Medium
	WT / *mms6*-GFP BPC	Δ*mamI* / *mms6*-GFP BPC	$< 10^{-5}$	****	0.9589	2	Large
	WT / *mms6*-GFP NBPC	Δ*mamL* / *mms6*-GFP NBPC	$< 10^{-5}$	****	0.4136	3	Large
	WT / *mms6*-GFP BPC	Δ*mamL* / *mms6*-GFP BPC	$< 10^{-5}$	****	0.9396	2	Large
	WT / *mms6*-GFP NBPC	Δ*mamN* Δ*mamO* / *mms6*-GFP NBPC	$< 10^{-5}$	****	0.3577	2	Large
	WT / *mms6*-GFP BPC	Δ*mamN* Δ*mamO* / *mms6*-GFP BPC	$< 10^{-5}$	****	0.9834	3	Large
Supplementary Figure S6B	ΔMAI ΔMIS / *mms6*-GFP NBPC	ΔMAI ΔMIS / $mms6_{\Delta NTD}$-GFP NBPC	$< 10^{-5}$	****	0.9872	1	Large
	ΔMAI ΔMIS / *mms6*-GFP BPC	ΔMAI ΔMIS / $mms6_{\Delta NTD}$-GFP BPC	$< 10^{-5}$	****	0.9402	1	Large
Supplementary Figure S8B	WT / *mms6*-GFP NBPC	WT / *mms6*-GFP BPC	$< 10^{-5}$	****	0.6677	2	Large
	WT / *mmsF*-GFP NBPC	WT / *mmsF*-GFP BPC	$< 10^{-5}$	****	0.08813	3	Small
	WT / *mmsF*-GFP NBPC	ΔMAI ΔMIS / *mmsF*-GFP NBPC	$< 10^{-5}$	****	0.8116	3	Large
	WT / *mmsF*-GFP BPC	ΔMAI ΔMIS / *mmsF*-GFP BPC	$< 10^{-5}$	****	0.7927	3	Large
	WT / *mmsF*-GFP NBPC	Δ*mms6* / *mmsF*-GFP NBPC	$< 10^{-5}$	****	0.1386	3	Small
	WT / *mmsF*-GFP BPC	Δ*mms6* / *mmsF*-GFP BPC	$< 10^{-5}$	****	0.259	3	Medium
	WT / *mmsF*-GFP NBPC	Δ*mamO* ΔR9 / *mmsF*-GFP NBPC	$< 10^{-5}$	****	0.5948	3	Large
	WT / *mmsF*-GFP BPC	Δ*mamO* ΔR9 / *mmsF*-GFP BPC	$< 10^{-5}$	****	0.7644	3	Large
	WT / *mmsF*-GFP NBPC	Δ*mamN* / *mmsF*-GFP NBPC	$< 10^{-5}$	****	0.6834	3	Large
	WT / *mmsF*-GFP BPC	Δ*mamN* / *mmsF*-GFP BPC	$< 10^{-5}$	****	0.7738	3	Large
Supplementary Figure S9C	WT / *mamG*-GFP NBPC	Δ*mms6* / *mamG*-GFP NBPC	$< 10^{-5}$	****	0.2454	3	Medium
	WT / *mamG*-GFP BPC	Δ*mms6* / *mamG*-GFP BPC	$< 10^{-5}$	****	0.1891	3	Medium
	WT / *mamF*-GFP NBPC	Δ*mms6* / *mamF*-GFP NBPC	$< 10^{-5}$	****	0.1673	3	Small
	WT / *mamF*-GFP BPC	Δ*mms6* / *mamF*-GFP BPC	0.8683	NS	0.00329	2	Negligible
	WT / *mamC*-GFP NBPC	Δ*mms6* / *mamC*-GFP NBPC	$< 10^{-5}$	****	0.2068	3	Medium
	WT / *mamC*-GFP BPC	Δ*mms6* / *mamC*-GFP BPC	$< 10^{-5}$	****	0.1348	3	Small
	WT / *mamG*-GFP NBPC	WT / *mamG*-GFP BPC	0.00234	*	0.09648	3	Small
	WT / *mamF*-GFP NBPC	WT / *mamF*-GFP BPC	$< 10^{-5}$	****	0.1553	3	Small
	WT / *mamC*-GFP NBPC	WT / *mamC*-GFP BPC	0.0014	*	0.1003	3	Small

Supplementary Table S8. Statistical tests for supplementary figures. Fisher's exact test tests the same hypothesis as the chi-squared test of independence but is more accurate for sample sizes under 500 and in cases where at least one sample has a value of zero. Cramer's V is an effect size measurement that measures how strongly two categorical fields are associated.
